# Correlation between Magnetocaloric Properties and Magnetic Exchange Interaction in Gd_54_Fe_36_B_10−*x*_Si*_x_* Amorphous Alloys

**DOI:** 10.3390/ma16103629

**Published:** 2023-05-10

**Authors:** Huiyan Zhang, Jia Tan, Xue Zhang, Jiazhe Yan, Han Shi, Ye Zhu, Weizhong Cheng, Hailing Li, Weihuo Li, Ailin Xia

**Affiliations:** 1 Key Laboratory of Green Fabrication and Surface Technology of Advanced Metal Materials, Anhui University of Technology, Ministry of Education, Ma’anshan 243002, China; 2 Ma’anshan Shuntai Rare Earth New Materials Co., Ltd., Ma’anshan 243100, China; 3 School of Material Science and Engineering, Anhui University of Technology, Ma’anshan 243002, China; 4 Wuhu Technology and Innovation Research Institute, Anhui University of Technology, Wuhu 241003, China

**Keywords:** Gd-Fe-based amorphous alloys, magnetocaloric effect, magnetic transition behavior, molecular field theory, exchange interaction

## Abstract

Gd_54_Fe_36_B_10−*x*_Si*_x_* (*x* = 0, 2, 5, 8, 10) amorphous ribbons were fabricated by melt-spinning technique. Based on the molecular field theory, the magnetic exchange interaction was analyzed by constructing the two-sublattice model and deriving the exchange constants *J*_GdGd_, *J*_GdFe_ and *J*_FeFe_. It was revealed that appropriate substitution content of Si for B can improve the thermal stability, maximum magnetic entropy change and widened table-like magnetocaloric effect of the alloys, while excessive Si will lead to the split of the crystallization exothermal peak, inflection-like magnetic transition and deterioration of magnetocaloric properties. These phenomena are probably correlated to the stronger atomic interaction of Fe-Si than that of Fe-B, which induced the compositional fluctuation or localized heterogeneity and then caused the different way of electron transfer and nonlinear variation in magnetic exchange constants, magnetic transition behavior and magnetocaloric performance. This work analyzes the effect of exchange interaction on magnetocaloric properties of Gd-TM amorphous alloys in detail.

## 1. Introduction

Magnetic refrigeration (MR) using solid magnetic material as a refrigerant has the advantages of environmental friendliness, low energy consumption and high efficiency. MR has been regarded as a potential alternative to replace traditional gas compression refrigeration [1]. The basic principle of MR is the intrinsic magnetocaloric effect (MCE) of magnetic materials: when a magnetic material is adiabatically magnetized, its total entropy *S* (*S* = *S*_M_ + *S*_E_ + *S*_L_, where *S*_M_, *S*_E_ and *S*_L_ denote magnetic entropy, electron entropy and lattice entropy, respectively) retains unchanged; the spin will align parallel to the direction of the applied magnetic field, inducing a decrease in *S*_M_ as well as an increase in *S*_E_ and *S*_L_, and therefore the enhanced lattice vibration leads to an increase in temperature [2]. The process is reversible during demagnetization. Usually, the isothermal magnetic entropy change Δ*S*_M_ or adiabatic temperature change Δ*T*_ad_ is utilized to estimate the magnitude of the magnetocaloric effect [3].

Magnetocaloric materials can be classified into first-order magnetic transition (FOMT) and second-order magnetic transition (SOMT) materials, according to the order of ferromagnetic (FM)–paramagnetic (PM) phase transition. FOMT material has a discontinuous magnetic transition process with temperature, which is usually related to the giant magnetocaloric effect (GMCE) [3]; however, narrow operating temperature span, high thermal and magnetic hysteresis, and inferior mechanical stability restrict its practical application. The mainly studied FOMT materials for near-room-temperature MR are Gd_5_(Si, Ge)_4_ alloys [4], La(Fe, Si)_13_ compounds [5], Mn-Fe-P-Si alloys [6], Heusler alloys [7], etc. In contrast, although the MCE of SOMT material is much lower, it usually possesses negligible magnetic hysteresis and nearly zero thermal hysteresis, which is beneficial for reducing energy losses in practical applications. Among the SOMT near-room-temperature refrigerants, Gd is the most representative as a reference material [8]. The high manufacturing cost and low corrosion resistance of Gd limit its practical application in near-room-temperature magnetic refrigeration technology [9].

Amorphous alloys with the unique atomic structure (short-range order and long-range disorder) belong to the SOMT material class in general and show excellent properties, such as neglectable magnetic and thermal hysteresis, tailorable Curie temperature, high mechanical properties and corrosion resistance, high resistivity (associated with low eddy current loss) and simple production process, which makes them good candidates as magnetic refrigerants [10]. Currently, studies on magnetocaloric amorphous alloys for the near-room-temperature region are mainly focused on Gd-based and Fe-based amorphous alloys. Although the Fe-based amorphous alloys have the advantages of suitable Curie temperature (*T*_C_) and low cost of raw material, their MCE is generally not high enough; oppositely, many of the Gd-based amorphous alloys exhibit large MCE, but their *T*_C_ is far from room temperature [11].

It has been revealed that the *T*_C_ of Gd-Co binary amorphous alloys can be raised from 166 K to 282 K by increasing the amount of Co due to the enhancement of Co-Co exchange interaction [12,13,14]. Additionally, further increment in the transition metals (TM) content through replacing Gd with Fe or Ni can promote the *T*_C_ to 310 K; however, the clusters or nanocrystals will appear more easily in the alloy, weakening the magnetocaloric properties of the alloys [15,16]. If the concentration of Gd remains constant, minor substitution of Fe or Ni for Co will improve or diminish the *T*_C_ of Gd-Co amorphous alloys owing to the variation in interaction between the transition metals [17,18]. Meanwhile, the peak value of Δ*S*_M_ (|Δ*S*_M_^pk^|) of the alloys nearly linearly depends on *T*_C_^−2/3^ [17,19]. However, in our previous work, it was found that excessive Fe replacement of Co results in a decrease in the *T*_C_ and |Δ*S*_M_^pk^| simultaneously [20], which may be ascribed to the enhancement of antiferromagnetic Gd-Fe exchange interactions and variation in predominant exchange interactions from ferrimagnetism to sperimagnetism [21,22].

On the basis of the molecular field theory (MFT), Gd-TM amorphous alloys can be constructed as a two-sublattice model, and three exchange interaction constants of *J*_GdGd_, *J*_TMTM_ and −*J*_GdTM_ (the positive and negative signs represent the ferromagnetic and antiferromagnetic exchange, respectively) can be derived by analyzing the temperature dependence of magnetization [23]. Particularly, in the Gd-Fe binary amorphous alloys, the change in Fe content causes the variation in magnetic transition behavior, which can be explained by the different exchange interaction constants [24]. Furthermore, Yano et al. reported that the addition of 10 at.% B in Gd_60_Fe_40_ amorphous alloy could manipulate the inversely bent (inflection-like) curve of the magnetization to the normal ferromagnetic curve, which is found to originate from the decrease in Fe magnetic moment and the enhancement of magnetic exchange constant |*J*_GdFe_| [25]. It has been demonstrated that co-doping of covalent B and Si modified the magnetic transition behavior and improved the magnetocaloric properties (with a larger |Δ*S*_M_| at higher working temperature) of the amorphous (Gd_0.6_Co_0.2_Fe_0.2_)_95_B_2_Si_3_ alloy [26]. In this work, a series of Gd_54_Fe_36_B_10−*x*_Si*_x_* (*x* = 0, 2, 5, 8, and 10) amorphous ribbons were prepared, and the influence of Si substitution for B on magnetic and magnetocaloric properties was investigated. In addition, to interpret the variation in magnetic and magnetocaloric properties with composition, the magnetic exchange interaction was analyzed based on the molecular field theory.

## 2. Experimental Details

Alloy ingots with nominal compositions of Gd_54_Fe_36_B_10−*x*_Si*_x_* (*x* = 0, 2, 5, 8, 10) were prepared by arc-melting mixtures of high-purity Gd (99.95 wt%) and Fe (99.95 wt%) metals and pre-alloy BFe and SiFe (mass ratios of B/Fe and Si/Fe were 17.62/81.46 and 22.25/74.53, respectively) under Ti-gettered argon atmosphere. Each ingot was overturned and remelted four times to ensure homogeneity. Then the as-spun ribbons were fabricated by single roller melt-spinning method with a copper wheel linear surface velocity of 50 m/s under a high-purity argon atmosphere. The structure of the as-spun ribbons was determined using an X-ray diffractometer (XRD, Bruker D8 Advance) in the 2*θ* range of 20°–80° with Cu K*_α_* radiation (λ = 0.154178 nm). Thermal analyses of the samples were carried out using a differential scanning calorimeter (DSC, Netzsch STA499 F3) under the protection of an argon gas flow at a heating rate of 0.33 K/s. A physical property measurement system (PPMS, Quantum Design PPMS Evercool-II) was adopted to measure the temperature dependence of magnetization (*M*-*T*) curves under the external magnetic field of 10 Oe and 6 kOe. A superconducting quantum interference device (SQUID, Quantum Design MPMS 3) was utilized to detect the isothermal magnetization (*M-H*) curves under an applied field change of 0–20 kOe at various selected temperatures in the vicinity of the magnetic transition temperature (*T*_tr_). All the magnetic properties were collected with the direction of the applied field parallel to the surface of the ribbons. To evaluate the magnetocaloric properties, the magnetic entropy change |Δ*S*_M_| was calculated from the *M-H* curves using the Maxwell equation as follows [27]:

(1) $$\Delta S_{M}(T,H)=S_{M}(T,H)-S_{M}(T,0)=\int_{0}^{H}{\left\{\frac{\partial M(T,H)}{\partial T}\right\}}_{H}dH$$

which indicates that the magnetic entropy change Δ*S*_M_(*T*,*H*) of a specific material is proportional to the derivative of magnetization with respect to temperature under a fixed field and to the magnetic field change. Typically, Equation (1) was numerically approximated as follows [6]:

(2) $$\Delta S_{M}(T,H)=\sum_{i}\frac{M_{i}(T_{n+1},H_{i})-M_{i}(T_{n},H_{i})}{T_{n+1}-T_{n}}\delta H_{i}$$

where *M_i_*(*T_n_*_+1_, *H_i_*) and *M_i_*(*T_n_*, *H_i_*) are experimental values of magnetization at temperatures *T_n_*_+1_ and *T_n_* under the applied field *H_i_*, respectively.

## 3. Results and Discussion

### 3.1. Characterization of Amorphous Structure

**Figure 1:**
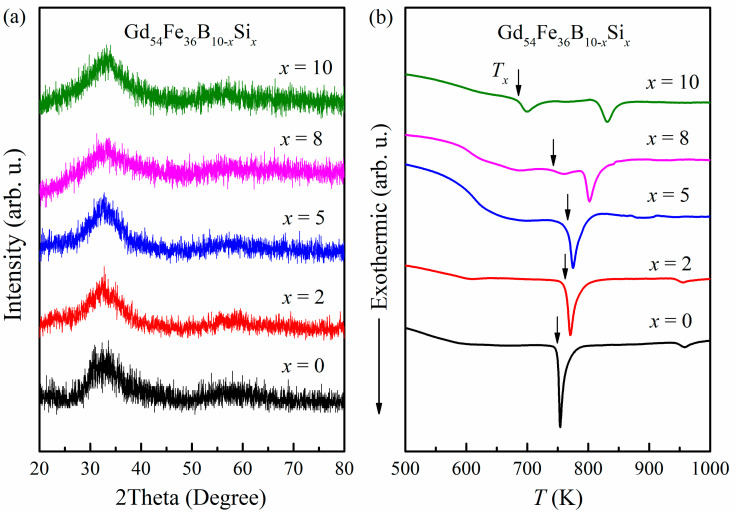
(**a**) XRD patterns and (**b**) DSC curves of the Gd_54_Fe_36_B_10−*x*_Si*_x_* as-spun ribbons.

Figure 1a shows the XRD patterns of the Gd_54_Fe_36_B_10−*x*_Si*_x_* (*x* = 0, 2, 5, 8, 10) as-spun ribbons. Only one broad diffraction peak at around 2*θ* = 33° without obvious peaks corresponding to the crystalline phase was obtained in each sample, which indicates the typical amorphous structure of the as-spun ribbons. The amorphous feature can be confirmed by the crystallization-related exothermic peaks in their DSC curves, as exhibited in Figure 1b, and the onset crystallization temperature (*T_x_*) of Gd_54_Fe_36_B_10−*x*_Si*_x_* amorphous alloys is 749, 762, 766, 743 and 686 K for *x* = 0, 2, 5, 8 and 10 respectively. With increasing content of Si, the *T_x_* increases firstly and then decreases, implying that appropriate co-addition of Si and B effectively enhanced the thermal stability of amorphous Gd_54_Fe_36_B_10−*x*_Si*_x_*, while immoderate Si content (*x* ≥ 8) induced the split of the exothermal peak and even two-step crystallization. There is no obvious glass transition in the DSC curves since the competing transformation to crystalline is predominant under the heating rate of 0.33 K/s [28]. For all the samples, the *T_x_* is high enough to ensure the amorphous structure near room temperature.

### 3.2. Determination of the Transition Temperature

**Figure 2:**
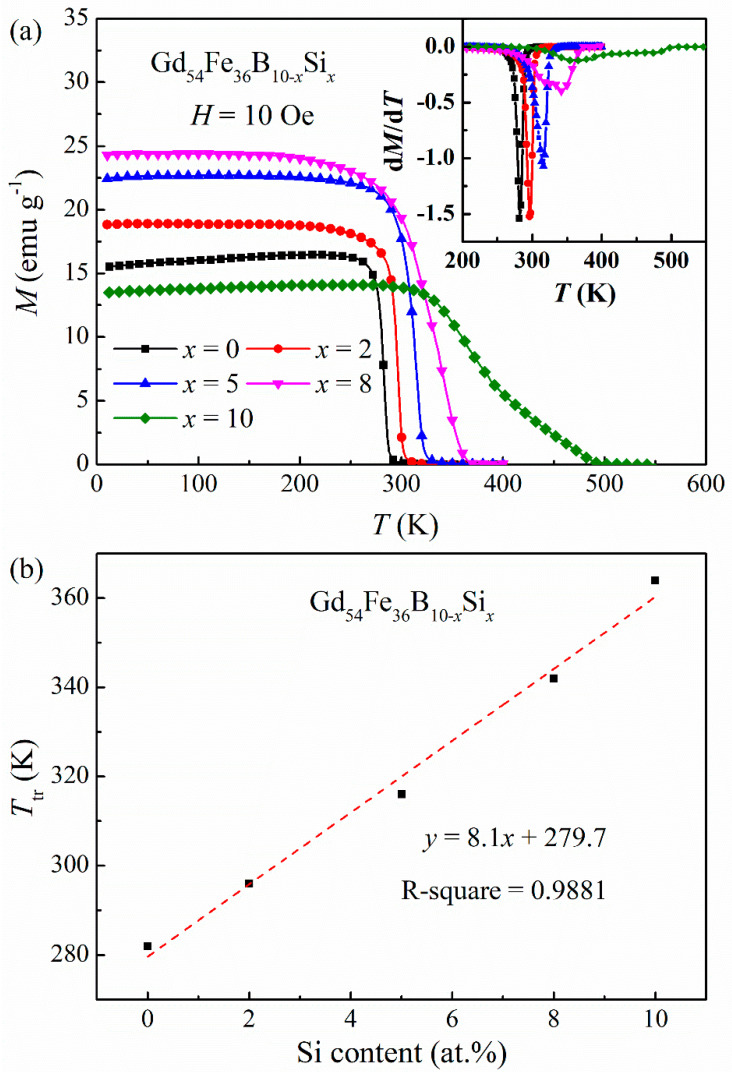
(**a**) Temperature-dependent magnetization curves of Gd_54_Fe_36_B_10−*x*_Si*_x_* amorphous ribbons under an applied field of 10 Oe. The inset presents the corresponding d*M/*d*T*-*T* plots. (**b**) Correlation between Si content and the *T*_C_ for Gd_54_Fe_36_B_10−*x*_Si*_x_* amorphous alloys.

Figure 2a shows the *M*-*T* curves of the Gd_54_Fe_36_B_10−*x*_Si*_x_* amorphous ribbons measured under the applied field of 10 Oe. It can be seen that the magnetization reduced with rising temperature, presenting a ferrimagnetic–paramagnetic transition. The magnetic transition temperature *T*_tr_ was determined by the inflection-point method, taking the temperature corresponding to the minimum derivative of *M*-*T* curve (namely the d*M*/d*T* vs. *T* plot, displayed in the inset of Figure 2a). For the samples with *x* = 0, 2, 5, 8 and 10, the *T*_tr_ is 282, 296, 316, 342 and 364 K, respectively. As illustrated in Figure 2b, the *T*_tr_ increased nearly linearly with increasing Si content for the Gd_54_Fe_36_B_10−*x*_Si*_x_* amorphous alloys, with the fitting expression of *T*_tr_ = 8.1*x* + 279.7, which is possibly due to the increase in magnetic exchange coupling [19,29,30]. Although only the concentration of non-magnetic B or Si elements changed, it may affect the magnetic moment and exchange interaction in the materials [25]. Similar results have been reported in amorphous alloys Gd_65_Fe_10_Co_10_Al_10_X_5_ (X = B, Si) and (Gd_0.6_Co_0.2_Fe_0.2_)_95_B*_x_*Si_5−*x*_ (*x* = 0, 2, 5) [26,31].

### 3.3. Magnetocaloric Properties

**Figure 3:**
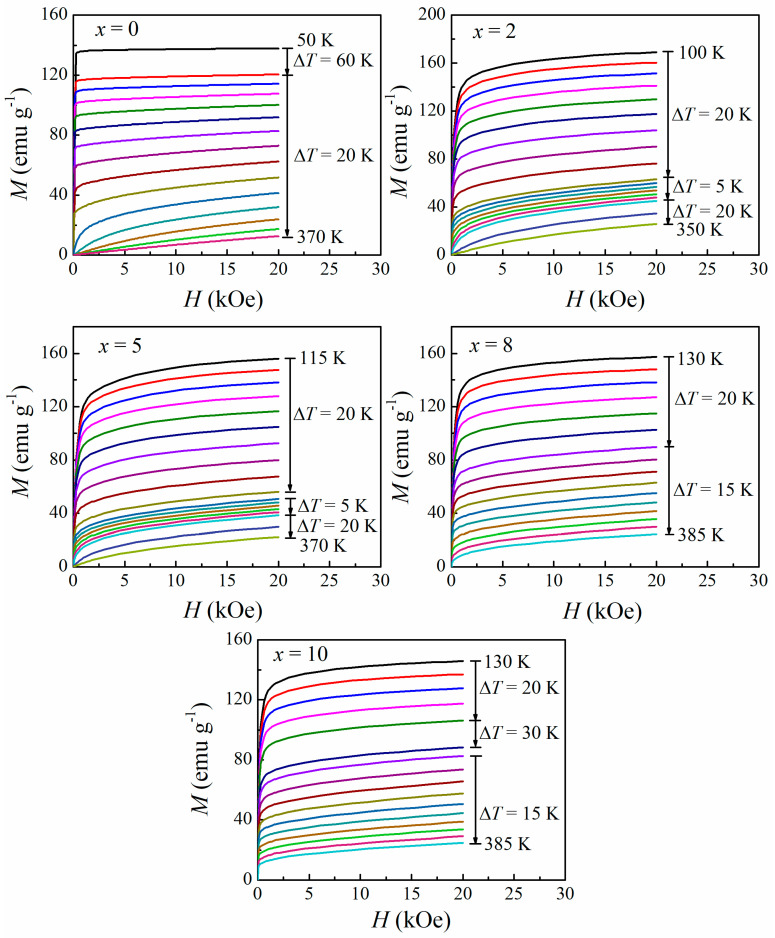
Isothermal magnetization curves of Gd_54_Fe_36_B_10−*x*_Si*_x_* amorphous ribbons near their *T*_C_ under the field changing from 0 to 20 kOe.

As indicated by Equation (1), the magnetic entropy change is approximately proportional to the d*M*/d*T*, and thereby the *M*-*H* isotherms of Gd_54_Fe_36_B_10−*x*_Si*_x_* (*x* = 0, 2, 5, 8, 10) amorphous alloys at different temperatures near their individual *T*_tr_ were measured under a magnetic field changing from 0 to 20 kOe, as exhibited in Figure 3. The sweeping rate of the field was slow enough to ensure that the data were recorded in an isothermal process. The obvious magnetic transition process near the *T*_tr_ could be observed in all the samples except Gd_54_Fe_36_Si_10_, which had not achieved a paramagnetic state at 385 K.

**Figure 4:**
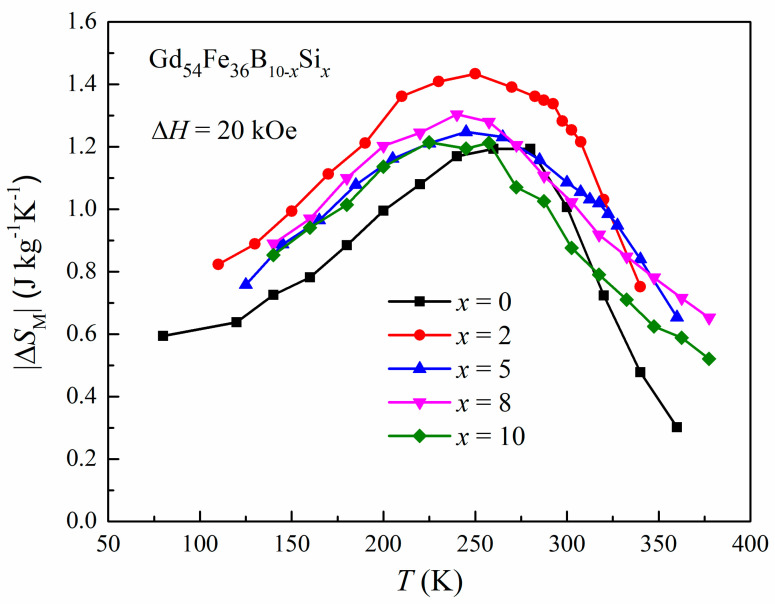
Temperature dependence of the |Δ*S*_M_| for Gd_54_Fe_36_B_10−*x*_Si*_x_* amorphous alloys under the magnetic field change of 20 kOe.

**Figure 5:**
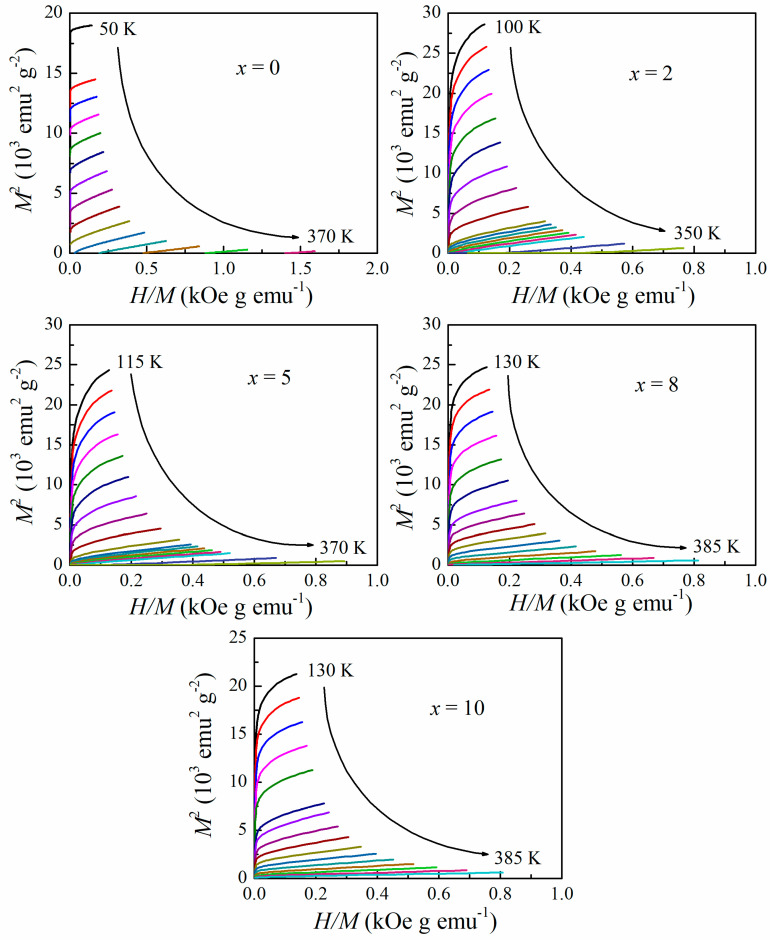
Arrott plots of amorphous Gd_54_Fe_36_B_10−*x*_Si*_x_* ribbons.

The magnetic entropy change as a function of temperature (|Δ*S*_M_| vs. *T* curves) is displayed in Figure 4. The maximum magnetic entropy change (|Δ*S*_M_^pk^|) of Gd_54_Fe_36_B_10−*x*_Si*_x_* amorphous alloys under field change of 20 kOe is 1.20, 1.43, 1.25, 1.30 and 1.21 J kg^−1^K^−1^ at 260, 250, 245, 240 and 225 K for *x* = 0, 2, 5, 8 and 10, respectively, which increases firstly and then decreases with increasing content of Si, showing a nonlinear correlation. Gd_54_Fe_36_B_8_Si_2_ possesses the highest |Δ*S*_M_^pk^|. In our previous study, a similar phenomenon was observed in the (Gd_0.6_Co_0.2_Fe_0.2_)_95_B*_x_*Si_5−*x*_ (*x* = 0, 2, 5) series of amorphous alloys [26]. The relative cooling power (*RCP*) is another parameter for estimating the MCE and energy efficiency of a magnetic refrigerant, and it can be evaluated by the product $RCP=|\Delta {S_{M}}^{\mathrm{pk}}|\times \Delta T_{\mathrm{FWHM}}$, where the Δ*T*_FWHM_ is the full width at half maximum of the entropy curve [32]. In this work, the values of *RCP* for the amorphous Gd_54_Fe_36_B_10−*x*_Si*_x_* with *x* = 0, 2, 5, 8 and 10 are 295, 374, 323, 362 and 321 J kg^−1^ (under the field change of 0–20 kOe), respectively. Owing to the larger value of |Δ*S*_M_^pk^|, the Gd_54_Fe_36_B_8_Si_2_ shows the highest *RCP*. Compared with the magnetocaloric properties (including |Δ*S*_M_^pk^| and *RCP*) of some other materials (with similar working temperature) listed in Table 1, the *RCP* values of the presently studied materials are higher in spite of their lower |Δ*S*_M_^pk^|, which results from the broadened entropy curve and larger Δ*T*_FWHM_. This is the typical characteristic of MCE obtained in Gd-based amorphous alloys with the second-order magnetic transition (SOMT) [18,19,29,30,33,34]. The Arrott plots (*M*^2^ vs. *H/M*) of Gd_54_Fe_36_B_10−*x*_Si*_x_* ribbons were derived from the *M*-*H* isotherms, as shown in Figure 5. According to Banerjee criteria [35], the slopes of the Arrott plots are positive in the whole temperature range for all the samples, indicating that their magnetic transition is SOMT.

**Figure 6:**
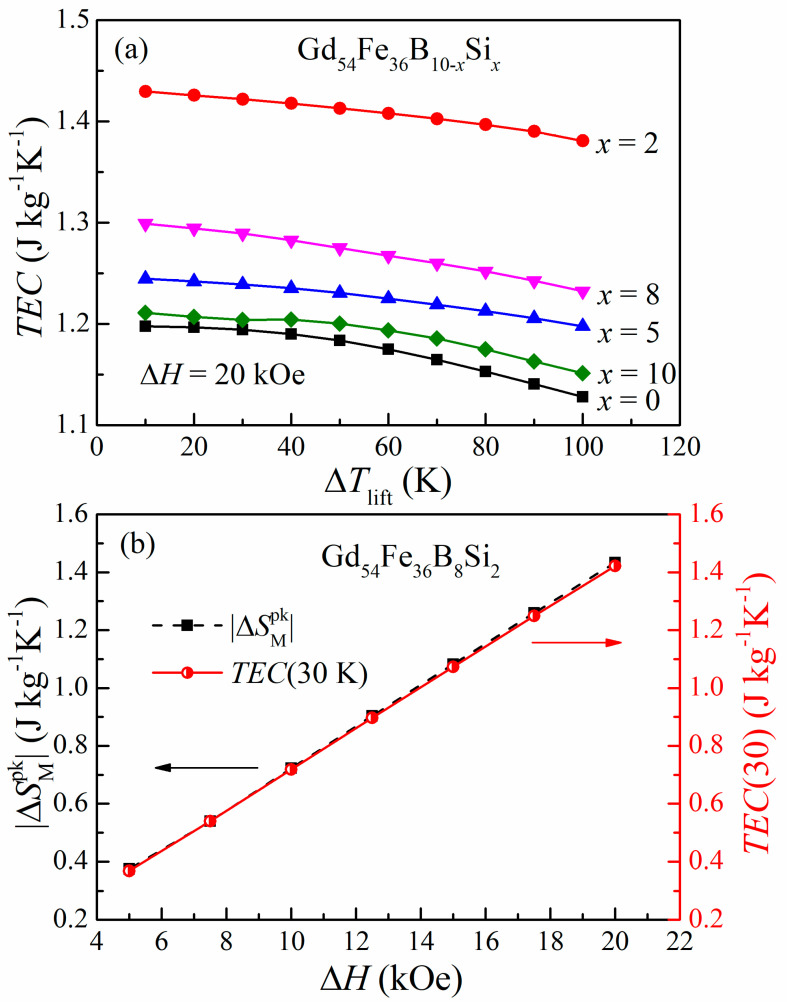
(**a**) *TEC* as a function of Δ*T_lift_* for the Gd_54_Fe_36_B_10−*x*_Si*_x_* amorphous alloys, under the field change of 0–20 kOe. (**b**) Magnetic field dependence of the *TEC*(30 K) and |Δ*S*_M_^pk^| for amorphous Gd_54_Fe_36_B_8_Si_2_.

However, the *RCP* is now recognized to overestimate the actual refrigerating capacity of the materials with a minor magnetic entropy change in an unreasonably broad temperature range [32]. In this regard, the temperature average entropy change (*TEC*) was introduced as a reliable figure of merit to assess the magnetocaloric efficiency; it is calculated by the following equation [36]:

(3) $$TEC(\Delta T_{lift})=\frac{1}{\Delta T_{lift}}\max\int_{T_{\mathrm{mid}}+\frac{\Delta T_{lift}}{2}}^{T_{\mathrm{mid}}-\frac{\Delta T_{lift}}{2}}|\Delta S_{M}(T)|dT$$

where Δ*T_lift_* is the desired lift temperature of the device and *T*_mid_ is the central temperature that maximizes the *TEC*(Δ*T_lift_*) value for a given Δ*T_lift_*. In this research, the Δ*T_lift_* was chosen between 10 K and 100 K with an interval of 10 K, and Figure 6a illustrates the variation in *TEC* values with respect to Δ*T_lift_* in the magnetic field change of 20 kOe for the Gd_54_Fe_36_B_10−*x*_Si*_x_* amorphous alloys. The correlation between *TEC* and the content of Si indicates that the partial replacement of B by Si improves the magnetocaloric performances in this series of materials, as reflected by the changing tendency of |Δ*S*_M_|. Additionally, the *TEC* values gradually decrease with increasing Δ*T_lift_* for each sample, and similar behavior has been reported previously [6,37,38]. It should be noted that the *TEC* changes very gently with the different Δ*T_lift_* values, which is ascribed to the table-like |Δ*S*_M_|(*T*) curves (the |Δ*S*_M_| retains almost constant in a wide temperature range). As revealed in Figure 6b, the *TEC*(30 K) and |Δ*S*_M_^pk^| of amorphous Gd_54_Fe_36_B_8_Si_2_ display a similar Δ*H* dependence, and their values are very close at any magnetic field, demonstrating less loss in the material during the magnetic transition. The obtained *TEC*(30 K, 20 kOe) and *TEC*(30 K, 15 kOe) values of Gd_54_Fe_36_B_8_Si_2_ are 1.42 J kg^−1^K^−1^ and 1.07 J kg^−1^K^−1^, respectively. Compared with some other materials, the values are lower than those of Gd (*TEC*(10 K, 10 kOe) = 2.91 J kg^−1^K^−1^) [36] but comparable to those of (La_0.7_Pr_0.3_)_0.8_Sr_0.2_Mn_0.9_Co_0.1_O_3±δ_ (*TEC*(10 K, 20 kOe) = 1.3 J kg^−1^K^−1^) [39] and La_0.65_Nd_0.05_Ba_0.3_Mn_0.85_Cr_0.15_O_3_ (*TEC*(25 K, 20 kOe) = 1.7 J kg^−1^K^−1^) [38] and higher than those of Fe_63.5_Cr_10_Si_13.5_B_9_Nb_3_Cu_1_ amorphous alloy (*TEC*(10 K, 15 kOe) = 0.83 J kg^−1^K^−1^) [36,38,39,40]. Although the magnetocaloric performance estimated by *TEC* is not very good, the table-like MCE with a wide temperature range was observed in all the samples, enabling them to be more suitable for the Ericsson thermodynamic cycle [32].

**Table 1:** Magnetocaloric properties (the magnetic transition temperature *T*_tr_, maximum magnetic entropy change |Δ*S*_M_^pk^| and its corresponding temperature *T*_pk_ and relative cooling power *RCP*) of amorphized Gd_54_Fe_36_B_10−*x*_Si*_x_* ribbons and some other materials in comparison. The A and C denote the amorphous and crystalline states, respectively.

Alloys	Structure	*T*_tr_ (K)	*T*_pk_ (K)	|Δ*S*_M_^pk^| (J kg^−1^K^−1^)	*RCP* (J kg^−1^)	Ref.
		*H* = 10 Oe	Δ*H* = 20 kOe			
Gd_54_Fe_36_B_10_	A	282	260	1.20	295	This work
Gd_54_Fe_36_B_8_Si_2_	A	296	250	1.43	374	This work
Gd_54_Fe_36_B_5_Si_5_	A	316	245	1.25	323	This work
Gd_54_Fe_36_B_2_Si_8_	A	342	240	1.30	362	This work
Gd_54_Fe_36_Si_10_	A	364	225	1.21	321	This work
Gd_50_Co_50_	A	267.2	~267	2.36	212.8	[33]
Gd_55_Co_35_Fe_10_	A	268	~268	1.72	337	[34]
Gd_50_Fe_45_Co_5_	A	289.5	288.5	1.85	289	[18]
Gd	C	292	~295	5.2	226.9	[9,41]
Gd_5_Si_2_Ge_2_	C	276	~276	18.4	195.3	[4,9]
LaFe_11.05_Co_0.91_Si_1.04_	C	282.1	~282	10.5	168.7	[5,9]
LaFe_11.40_Co_0.52_Si_1.09_	C	237.7	~238	16.8	147.4	[5,9]

### 3.4. Magnetic Exchange Interaction

In Figure 2, it can be found that the ferrimagnetic–paramagnetic magnetic transition process becomes broad and gentle with increasing Si content, which is possibly attributed to the variation in Fe magnetic moment and magnetic exchange constant (*J*_GdGd_, *J*_GdFe_, *J*_FeFe_) induced by the replacement of B with Si [25,42]. Additionally, the *T*_tr_ obtained in the low magnetic field of 10 Oe is higher than the *T*_pk_ achieved under the high magnetic field of 20 kOe, while the *T*_tr_ is similar to the *T*_pk_ for the other series of alloys, as displayed in Table 1. Especially for the present Si-containing Gd_54_Fe_36_B_10−*x*_Si*_x_* amorphous samples, the discrepancy between *T*_tr_ and *T*_pk_ becomes larger with increasing content of Si, and this may be caused by the varied antiferromagnetic coupling between Fe and Gd sublattices associated with the composition and magnetic field [24,25].

**Figure 7:**
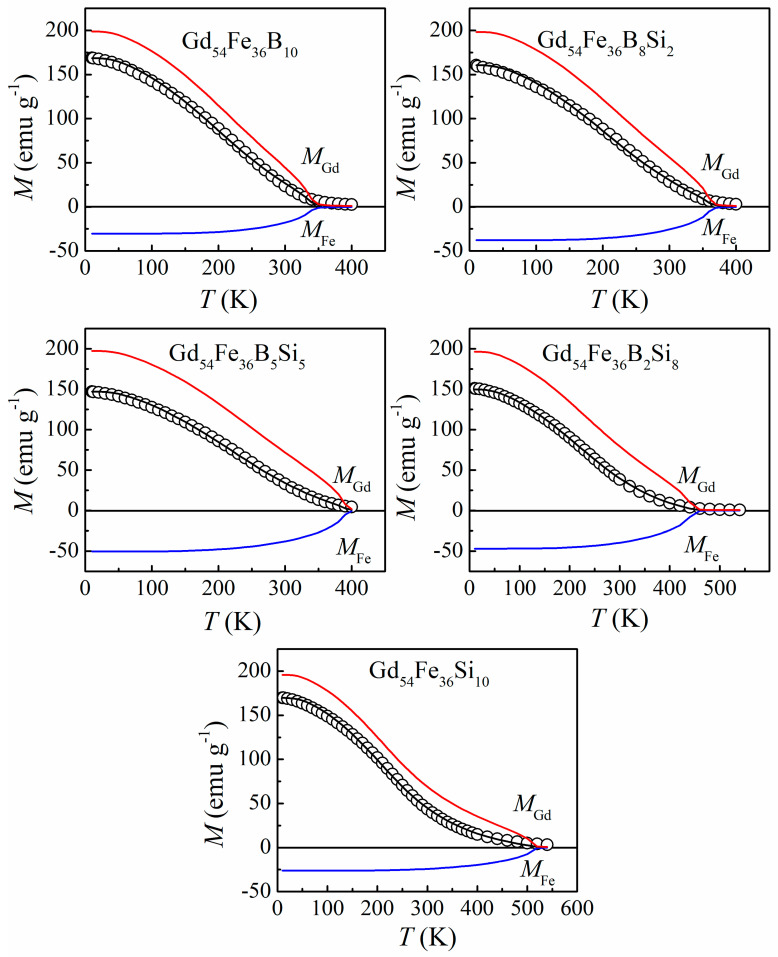
*M*-*T* curves for the Gd_54_Fe_36_B_10−*x*_Si*_x_* (*x* = 0, 2, 5, 8, 10) amorphous alloys under magnetic field of 6 kOe (open circles) and the fitting results (solid line). Calculated data of two sublattice magnetizations (*M*_Gd_ and *M*_Fe_ are denoted as “red line” and “blue line”, respectively) are also shown.

To investigate the origin of these phenomena in detail, an MFT analysis was carried out with the two-sublattice model [43]. First of all, the *M*-*T* curves of Gd_54_Fe_36_B_10−*x*_Si*_x_* amorphous ribbons were measured under the magnetic field of 6 kOe, which ensures the saturation state of the samples, as exhibited in Figure 7 (open circle). The inflection-like behavior with the characteristic of an inversely bent curve in a relatively wide temperature range can be observed for *x* = 8 and 10, similar to the transition type revealed in Gd-rich region Gd-Fe amorphous ribbons [25]. In the next step, each sublattice magnetization *M*_Gd_ and *M*_Fe_ and the total magnetization *M* were calculated by assigning some values to three exchange integration constants, *J*_GdGd_, *J*_GdFe_ and *J*_FeFe_, at a certain temperature *T*, then the *M*-*T* curves in the field of 6 kOe was fitted through adopting the nonlinear least square method [44]. The ferrimagnetic model was constructed with the following parameters: The Landé factors of Gd and Fe are *ց*_Gd_ = *ց*_Fe_ = 2. The coordination number *Z_ij_* (*i*, *j* = Gd, Fe) is expressed as *Z*_GdGd_ = *Z*_FeGd_ = 7.2 ($=\frac{12X_{\mathrm{Gd}}}{X_{\mathrm{Gd}}+X_{\mathrm{Fe}}}$) and *Z*_GdFe_ = *Z*_FeFe_ = 4.8 ($=\frac{12X_{\mathrm{Fe}}}{X_{\mathrm{Gd}}+X_{\mathrm{Fe}}}$), where *X*_Fe_ and *X*_Gd_ are atomic content of Fe and Gd respectively. The spin quantum number *S*_Gd_ is 7/2 for the Gd sublattice, while the *S*_Fe_ was derived from Fe magnetic moment *μ*_Fe_ (*μ*_Fe_ = *ց*_Fe_*S*_Fe_), and *μ*_Fe_ was evaluated from the magnetization *μ*_a_ at 10 K under 6 kOe by $\mu_{a}=|X_{\mathrm{Gd}}\mu_{\mathrm{Gd}}-X_{\mathrm{Fe}}\mu_{\mathrm{Fe}}|/100$ (where *μ*_Gd_ = *ց*_Gd_*S*_Gd_ = 7 *μ*_B_) [45]. As a result, the *J*_GdGd_, *J*_GdFe_ and *J*_FeFe_ were derived, and the fitting profiles of *M*_Gd_, *M*_Fe_ and *M* are depicted in Figure 7 (solid line). It can be seen that all the calculated results are in good accordance with the experimental data.

**Figure 8:**
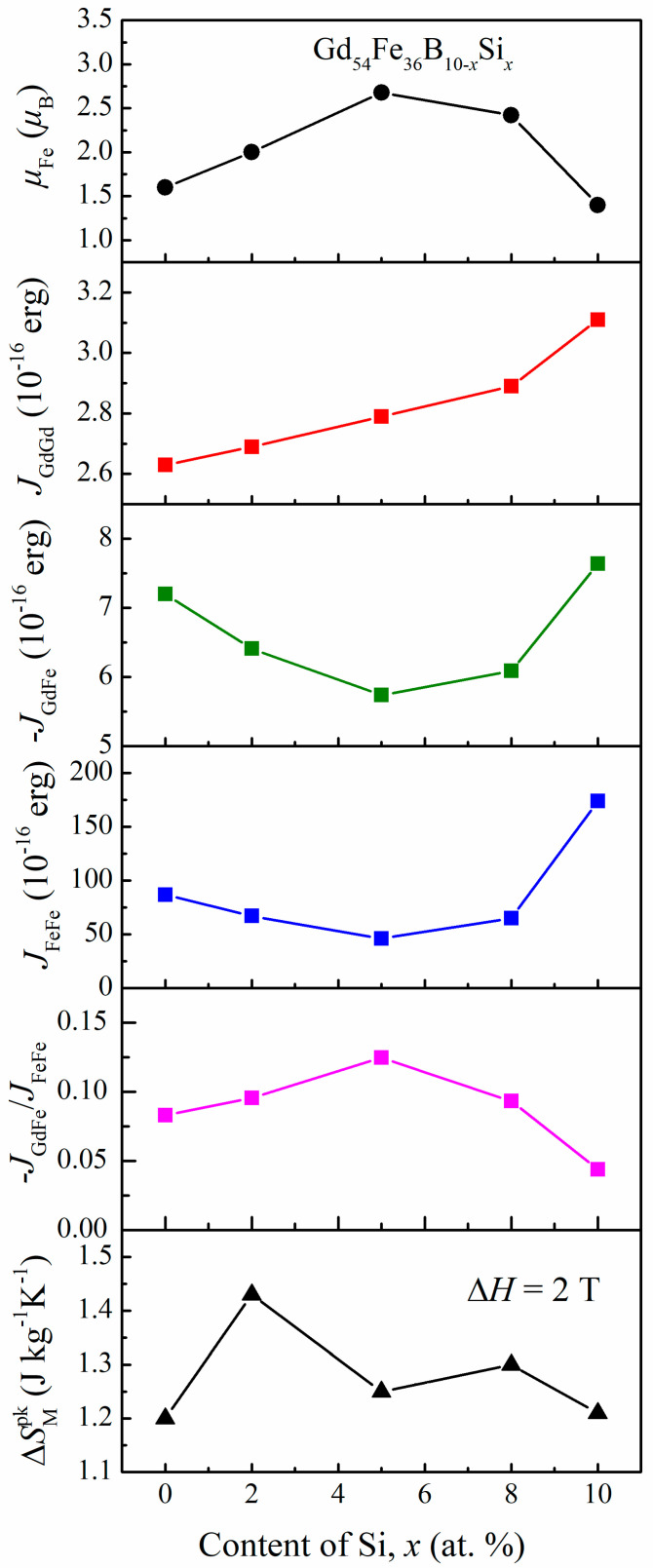
Correlation of magnetic moment of Fe, exchange interaction constants *J*_GdGd_, −*J*_GdFe_ and *J*_FeFe_, ratio of −*J*_GdFe_/*J*_FeFe_, and |Δ*S*_M_^pk^| with the content of Si for amorphous Gd_54_Fe_36_B_10−*x*_Si*_x_*. The sign of *J*_GdGd_ and *J*_FeFe_ is positive but *J*_GdFe_ is negative.

The content dependence of *μ*_Fe_, *J*_GdGd_, −*J*_GdFe_, *J*_FeFe_, the −*J*_GdFe_/*J*_FeFe_ ratio and |Δ*S*_M_^pk^| for Gd_54_Fe_36_B_10−*x*_Si*_x_* amorphous alloys is displayed in Figure 8. The *μ*_Fe_ increases from 1.60 *μ*_B_ (for *x* = 0) to 2.68 *μ*_B_ (for *x* = 2) first and then decreases to 1.4 *μ*_B_ (for *x* = 10). Similar results can be found in amorphous alloys Fe_56_Gd_24_Si_12_B_8_ (*μ*_Fe_ ≈ 1.40 *μ*_B_) and Fe_56_Gd_24_B_20_ (*μ*_Fe_ = 1.22 *μ*_B_) [45,46]. On one side, compared with the B element, Si possesses more covalent electrons (mainly 3p electrons), which possibly intensifies the transfer of electrons to the 3d orbital of Fe, leading to the lower value of *μ*_Fe_ [25,46,47]. On the other side, the substitution of Si for B could change the local environment and affect the magnetic moment of Fe atoms [48]. In this study, for the moderate Si content, B may absorb electrons from Fe atoms and promote *μ*_Fe_ [49]; for the alloys with high content of Si or B (*x* = 0 and 10), the p-d hybridization dominates the reduction in *μ*_Fe_ [50].

With the replacement of B by Si in amorphous Gd_54_Fe_36_B_10−*x*_Si*_x_* alloys, the *J*_GdGd_ increases slightly, reflecting the decrease in the average distance between Gd atoms [24], which is probably attributed to the stronger atomic interaction between Fe and Si (in comparison with the Fe-B pairs), and more metalloid atoms tend to surround Fe atoms [51]. Furthermore, after the addition of Si, fewer B atoms appear at the nearest neighbor locations around Fe atoms, and as described above, B absorbs electrons from Fe for the lower B content [52].

It can be seen that the introduction of Si has stronger impacts on −*J*_GdFe_ and *J*_FeFe_ than on *J*_GdGd_. Additionally, both −*J*_GdFe_ and *J*_FeFe_ decrease firstly and then increase with increasing Si content; the variation rule is opposite to that of *μ*_Fe_, as displayed in Figure 8. The overlap between 5d electron wave-functions of Gd and 3d electron wave-functions of Fe is considered to be the origin of the antiferromagnetic exchange coupling −*J*_GdFe_ [53]; therefore, the lost 3d electron of Fe absorbed by the surrounding B (with moderate content of Si, i.e., *x* = 2, 5 and 8) is in accordance with the weakened 3d–5d interaction [25]. The effect of B addition on *J*_FeFe_ in the amorphous Gd_54_Fe_36_B_10_ alloys is neglectable [25], which can be interpreted by its statistically random distribution in the structure [24]. However, Si has a stronger atomic interaction with Fe and then preferentially neighbors Fe, resulting in the deviation from the statistical distribution, which is reflected by the great increase in exchange constant *J*_FeFe_ in the Gd_54_Fe_36_Si_10_ [24]. For the Gd_54_Fe_36_B_10−*x*_Si*_x_* samples, partial replacement of B by Si may reduce the distance between Fe atoms, and the exchange interaction decreases according to the Bethe–Slater curve [54].

It has been reported that the inflection-like magnetic transition of a Gd-TM amorphous alloy can be adjusted to normal ferromagnetic–paramagnetic transition by increasing the ratio of −*J*_GdTM_/*J*_TMTM_ when the *J*_GdGd_ remains constant [55]. As shown in Figure 8, the value of −*J*_GdFe_/*J*_FeFe_ increases first and then decreases with Si content rising from 0 to 10, which can explain the inflection-like *M*-*T* curves observed for Gd_54_Fe_36_B_10−*x*_Si*_x_* amorphous alloys with *x* = 8 and 10. Moreover, adding more Si probably makes the atomic structure deviate from the statistical distribution, which leads to compositional fluctuation or localized heterogeneity [24]; then the larger discrepancy between *T*_tr_ and *T*_pk_ was obtained [56].

In Figure 8, the variation tendency of −*J*_GdFe_/*J*_FeFe_ and |Δ*S*_M_^pk^| with Si content is similar, except for the Gd_54_Fe_36_B_5_Si_5_; the atomic-scale structure and accurate *μ*_Fe_ of this series of alloys need to be clarified further. Nevertheless, it can be revealed that the appropriate substitution of Si for B promotes the value of |Δ*S*_M_^pk^| and table-like MCE of amorphous Gd_54_Fe_36_B_10−*x*_Si*_x_* alloys; this can be attributed to the enhancement of the −*J*_GdFe_/*J*_FeFe_ and modified magnetic transition behavior. An excessive amount of Si results in a decline in the −*J*_GdFe_/*J*_FeFe_, an inflection-like transition and the deterioration of magnetocaloric properties.

## 4. Conclusions

In summary, the effect of Si substitution for B on thermal stability, magnetic transition behavior and magnetocaloric properties of melt-spun Gd_54_Fe_36_B_10−*x*_Si*_x_* (*x* = 0, 2, 5, 8, 10) amorphous alloys was researched. With appropriate content of Si, the alloys showed enhanced thermal stability and broadened table-like MCE; with excessive Si, the alloys exhibited poorer thermal stability, inflection-like transition behavior and weakened MCE. Among present alloys, Gd_54_Fe_36_B_8_Si_2_ possesses the largest values of |Δ*S*_M_^pk^| (1.43 J kg^−1^K^−1^), *RCP* (374 J kg^−1^) and *TEC*(30 K) (1.42 J kg^−1^K^−1^) under an applied field of 20 kOe, as well as a table-like MCE, which makes it more suitable for the MR with the Ericsson cycle.

The variation in magnetic exchange constants *J*_GdGd_, *J*_GdFe_ and *J*_FeFe_ was obtained by fitting the temperature dependence of magnetization according to the molecular field theory and two-sublattice model. Substitution of B with Si induces the different ways of electron transfer and different atomic interaction (Fe-Si pairs are stronger than Fe-B), resulting in the nonlinear correlation between *μ*_Fe_, *J*_GdGd_, −*J*_GdFe_, *J*_FeFe_ and −*J*_GdFe_/*J*_FeFe_ and Si content. Therefore, the shape of the magnetic transition curve and the magnetocaloric properties changed nonlinearly.

## Data Availability

The data presented in this study are available on request from the corresponding author.
